# Laparoscopic assisted low anterior resection for advanced rectal cancer in a kidney transplant recipient: A case report

**DOI:** 10.1097/MD.0000000000005198

**Published:** 2016-11-04

**Authors:** Zenan Xia, Weijie Chen, Ru Yao, Guole Lin, Huizhong Qiu

**Affiliations:** a Department of Surgery, Peking Union Medical College Hospital, Chinese Academy of Medical Sciences; b Department of General Surgery, Peking Union Medical College Hospital, Chinese Academy of Medical Sciences, Beijing, China.

**Keywords:** case report, kidney transplantation, laparoscopic surgery, low anterior resection, rectal cancer

## Abstract

**Introduction:**

Development of de novo malignancy has become a major cause of late mortality in solid organ transplant recipients. Surgery is currently the most important treatment of choice for transplant patients with resectable CRC. However, conventional open surgery represents a great risk to these high-risk patients. They seem to benefit more from laparoscopic surgery, based on the favorable oncological outcome and remarkable short-term advantages of this approach.

**Patient concerns:**

In this study, we have reported a case of a 50-year-old man who had underwent kidney transplantation for 4 years. He presented with recurrent hematochezia and frequent loose stools for 1 year, and consulted a doctor for recent progressive general malaise and weight loss.

**Diagnoses:**

Colonoscopy revealed a near-circumferential mass at the middle rectum about 8 cm from anal verge. Further biopsy confirmed a diagnosis of adenocarcinoma. Following computed tomography demonstrated peripheral lymph node metastasis, but no signs of distant metastasis.

**Interventions:**

The patient underwent a laparoscopic assisted low anterior resection with total mesorectal excision for rectal cancer. Concomitantly, a loop transverse colostomy was performed to prevent anastomotic leakage. The surgery was completed within 120 min with a blood loss of 100 mL, and immunosuppressive therapy was not stopped perioperatively. Considering the tumor stage of pT3N1M0, the patient also received adjuvant chemotherapy with a regimen of FOLFOX for 8 cycles.

**Outcomes:**

Anastomotic bleeding occurred in this patient about 4 h after surgery, and a control of hemorrhage per anus was performed timely. The following postoperative course was uneventful without any complications, and graft function stayed well. After 4 months of follow-up period, the patient was in a good condition. No evidences of local recurrence and distant metastasis were found.

**Conclusion:**

We have presented a case of successful laparoscopic resection for advanced rectal cancer in a kidney transplant recipient. We believe laparoscopic surgery for CRC in transplant recipients is technically feasible and oncologically safe, which could be a preferred option of surgical procedure in the near future.

## 1 Introduction

Colorectal cancer (CRC) is one of the most common malignancies and the leading causes of cancer-related death worldwide.^[1]^ Surgery is the cornerstone of curative treatment for patients with resectable CRC. A number of earlier randomized trials and meta-analyses confirmed that laparoscopic surgery for CRC was not inferior to open surgery in terms of survival and recurrence rates, but provides significant short-term advantages, including a shorter hospital stay, reduced analgesic use, faster recovery of intestinal function, and earlier return to activities of daily life.^[2,3]^ Therefore, laparoscopic-assisted surgery has been widely accepted as an alternative to conventional open surgery for CRC.^[4]^

Currently, due to the development of minimally invasive techniques, the indications for laparoscopic surgery have gradually expanded to high-risk patients with CRC.^[5]^ Recipients of solid organ transplantation not only have associated problems of chronic immunosuppression and allograft dysfunction, but also suffer from numerous comorbidities, as the primary etiology of their organ failure.^[6,7]^ Conventional surgical treatment for CRC represents a great risk to these high-risk patients, and the benefits of minimal access surgery seem to be shared by them.^[8]^ However, there is still some lack of literature about the evaluation on outcome of laparoscopic surgery for CRC in patients after organ transplantation. Herein, we report a case of a patient who presented with advanced rectal cancer 4 years after kidney transplantation, and underwent laparoscopic assisted low anterior resection.

## 2 Case report

A 51-year-old Chinese male had undergone living-donor kidney transplantation for end-stage renal disease (ESRD) due to 8 years of nephrotic syndrome in 2011 at the age of 46 years. His immunosuppression regimen included a combination therapy with tacrolimus 2.5 mg/d, mycophenolate mofetil 1.5 g/d, and prednisolone 10 mg/d. The blood concentration of tacrolimus (FK-506) was monitored regularly at a target level of 4 to 10 ng/mL. No colonoscopy had been performed prior to transplantation, and he did not receive colonoscopic surveillance postoperatively. Renal graft function remained stable in him without any rejection episodes.

Four years after transplantation, the patient presented with recurrent hematochezia (bright red blood per rectum mixed with stools), and a change in bowel habits manifested by frequent loose stools. He did not pay attention to these abnormal conditions until he began to suffer from progressive general malaise and weight loss of 3 kg during the period of 1 month. A colonoscopy was taken subsequently in January 2016, which revealed a near-circumferential mass at the middle rectum about 8 cm from anal verge accompanied by moderate luminal stenosis. The friable lesion proved to be adenocarcinoma by further biopsy. Having made a definite diagnosis, the patient was admitted to our institution in February 2016. He had a previous history of hypertension for 7 years that could be generally controlled by medication, and cigarette smoking for more than 30 years. Regarding his family history, there were no remarkable findings.

**Figure 1:**
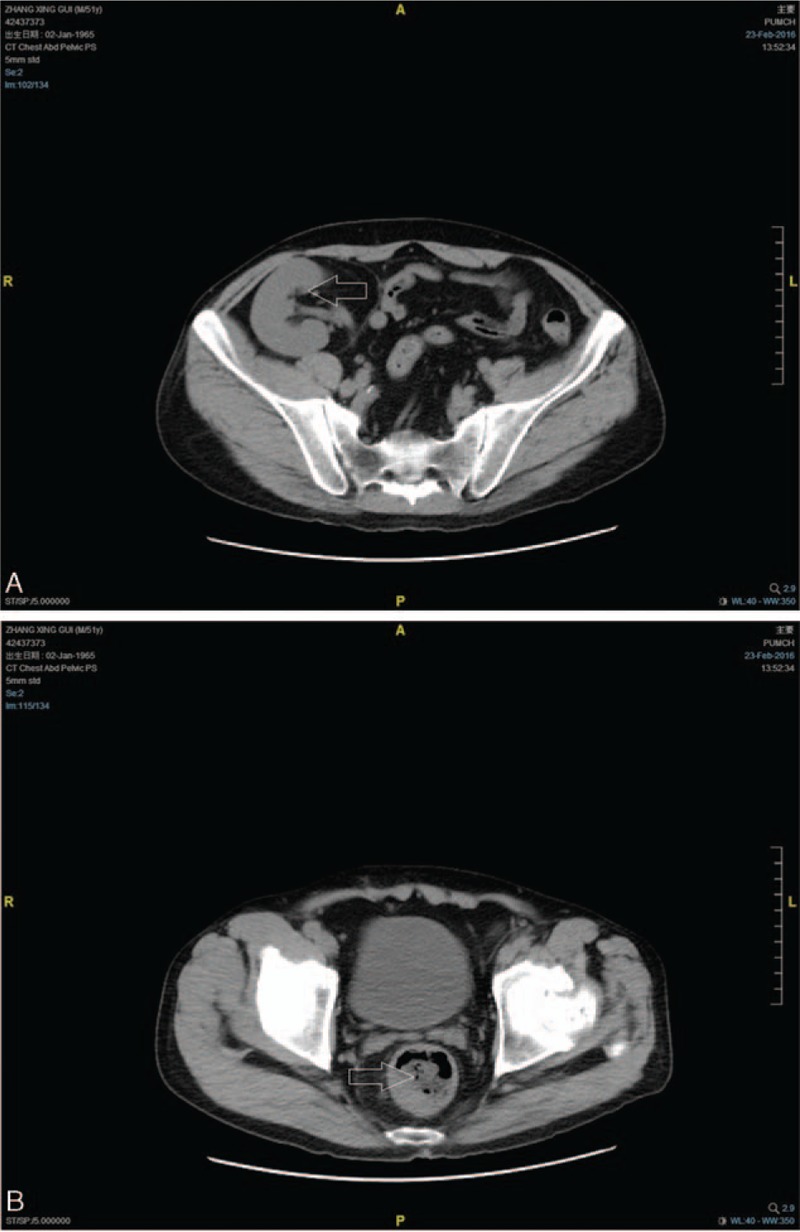
Computed tomography scan showed a soft tissue density mass (arrow) protruding into the lumen of rectum (A) and a transplant kidney (arrow) in the right pelvic cavity (B).

On admission, detailed physical examination revealed a temperature of 36.5°C, pulse of 80 bpm, and blood pressure of 106/60 mm Hg. No special signs were noted except for a healed surgery scar on his right lower abdominal wall. The lower margin of a solid, irregular mass was touched about 6 cm from anal verge through a digital rectal examination. Laboratory tests showed hemoglobin was 133 g/L, fecal occult blood test was positive, serum creatinine was 88 μmol/L, liver function test was normal, and most serum tumor markers (AFP, CEA, CA19-9, and CA72-4) were within the normal range but CA242 was slightly elevated. The following computed tomography (CT) scan demonstrated a soft tissue density mass protruding into the lumen of upper-middle rectum, with the wall thickening and peripheral lymph node metastasis and the transplant kidney was located in the right pelvic cavity (Fig. 1). No evidences of distant metastasis were suggested. FK-506 was 4.7 ng/mL at that time. According to recommendation of interdisciplinary team including urologists, medical physicians, and general surgeons, immunosuppressive therapy with previous regimen was extended perioperatively. Meanwhile, 100 mg of intravenous hydrocortisone was added before anesthesia induction, and turned to a dose of 50 mg/8 h postoperatively for 1 day to prevent acute renal failure caused by the attack of surgery.

**Figure 2:**
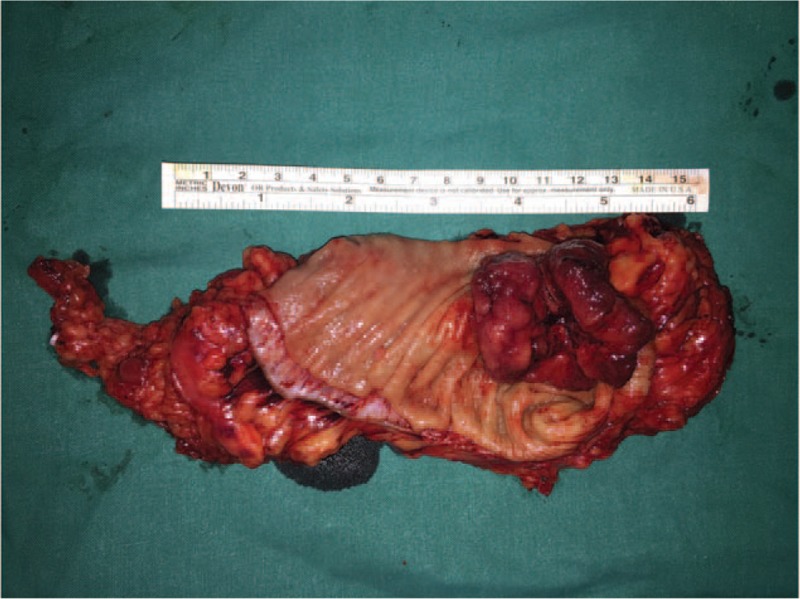
A tumor measuring 4.2 × 5 cm accompanied with rectum and the surrounding mesorectum were removed. Distance of the tumor from the distal resection margin was 3 cm and from the proximal resection margin was 10 cm.

**Figure 3:**
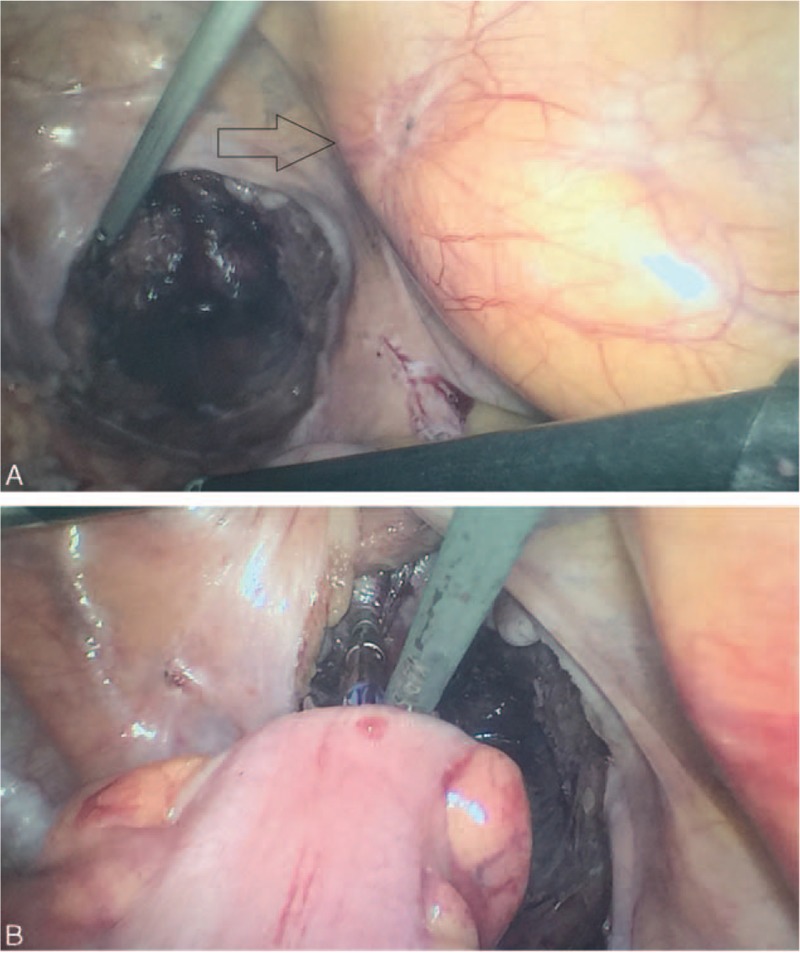
Laparoscopic assisted resection for rectal cancer. (A) Transection of distal rectum near the graft (arrow). (B) Colorectal anastomosis using a transanal circular stapler.

**Figure 4:**
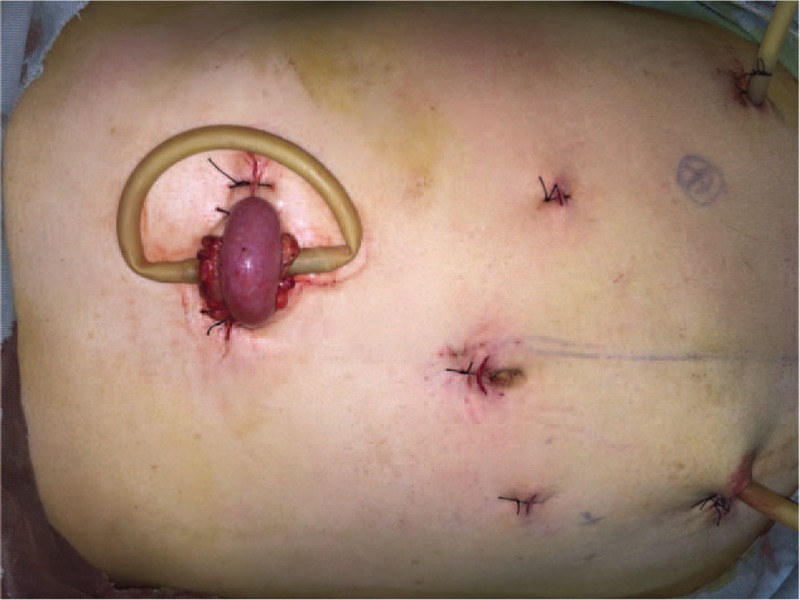
A loop transverse colostomy was performed to prevent postoperative anastomotic leakage.

**Figure 5:**
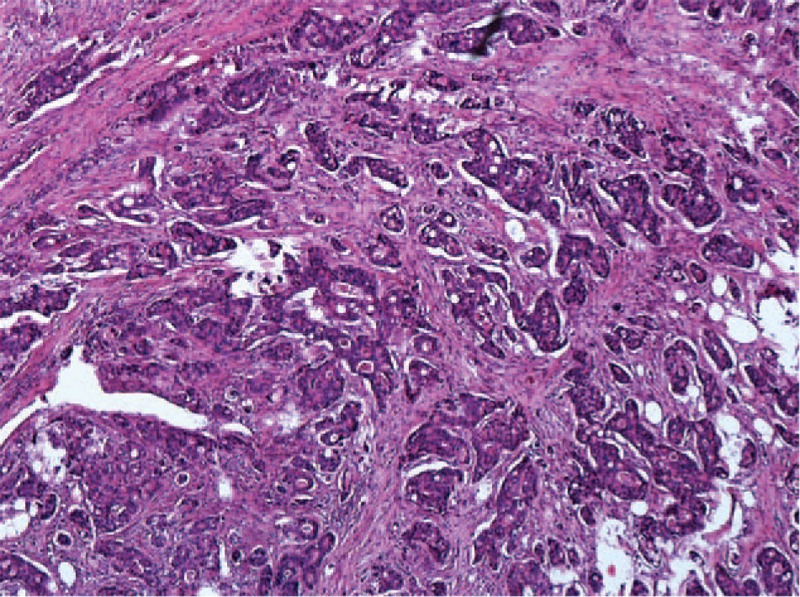
Pathology showed a well-moderately differentiated adenocarcinoma with invasion through the muscularis propria into pericolorectal tissues.

Without any surgery contraindications, a laparoscopic assisted low anterior resection with total mesorectal excision (TME) was performed then. After induction of general anesthesia, the patient was positioned in the lithotomy position. Complete exploration of the abdominal cavity suggested that there was no liver and peritoneal carcinomatosis. The tumor was identified locating at the middle-lower rectum. Specific surgical procedures started with lysis of abdominal adhesions from previous surgery. The sigmoid mesocolon and mesorectum were dissected along the inner side of the ureters by harmonic scalpel, and the vessels and lymphatics were ligated at the root of the superior rectal vessel with Hem-o-lock. The distal rectum was transected intracorporeally 3 cm from the distal margin of tumor with a EC45-A laparoscopic linear stapler (Johnson & Johnson, Cincinnati, OH, USA). After that, a 6 cm left supraumbilical incision was made to remove the proximal rectum, the distal sigmoid colon, and the surrounding tissues of the rectum 10 cm from the proximal margin of tumor. The specimen was obtained for further pathological evaluation (Fig. 2). Reconstruction was performed intracorporeally in the manner of straight end-to-end colorectal anastomosis using a 28 mm transanal circular stapler (COVIDIEN, Mansfield, MA, USA) (Fig. 3). Finally, 2 drainage tubes were placed in the pelvic cavity surrounding the anastomosis site. In order to prevent postoperative anastomotic leakage, a loop transverse colostomy was performed (Fig. 4). The operation was completed successfully within 2 h with a proximate blood loss of 100 mL. Histopathology revealed a well-moderately differentiated adenocarcinoma measuring 4.2 × 5 × 2.3 cm, with invasion through the muscularis propria into pericolorectal tissues (Fig. 5). The resection margins were free of tumor. Among the removed 14 lymph nodes, 3 contained metastatic cancer, indicating the tumor stage of pT3N1M0. Furthermore, immunohistochemistry showed the tumor was negative for MLH-1, MLH-2, MLH-6, and PMS-2.

Approximately 4 h after surgery, the patient developed archorrhagia (dark red blood and clots per rectum), and the amount of blood loss increased up to 200 mL within 1 h. After exploration per anus, a bleeding wound near the anastomosis was identified and the control of hemorrhage was performed timely. There was no recurrence of archorrhagia ever since. The further postoperative course was uneventful. Graft function stayed well, and serum creatinine levels were always within the normal limits, ranging from 80 to 104 μmol/L. The ostomy was opened on the third postoperative day, and flatus was passed then. The patient went on a liquid diet 4 days after surgery and made a recovery soon. He stayed in hospital for 9 days after surgery, and drainage tubes were removed at discharge.

During 4 months of follow-up period, immunosuppressive therapy with tacrolimus 2.5 mg/d, mycophenolate mofetil 1.5 g/d, and prednisolone 10 mg/d continued, no allograft rejection and complications were observed. Considering his tumor stage, he received adjuvant chemotherapy with a regimen of FOLFOX (oxaliplatin/5-fluorouracil/calcium folinate). After 8 cycles of chemotherapy, repeated CT scan indicated no evidences of local recurrence and distant metastasis. To date, the patient was in a good condition.

## 3 Discussion

We have reported the detailed case of a patient after kidney transplantation, in whom laparoscopic surgery for advanced rectal cancer had been performed. Our case demonstrates that laparoscopic assisted surgery for advanced rectal cancer can be tolerated by kidney transplant recipients, and its short as well as long-term outcomes are acceptable and encouraging.

Kidney transplantation is a definite treatment for patients with ESRD. As surgical techniques, organ procurement, immunosuppression regimen, and postoperative monitoring have improved, kidney transplant recipients have a higher treatment success rate and a longer life span.^[9]^ However, the development of de novo malignancy has become a major cause of late mortality in these patients.^[10]^

The increased risk of CRC after kidney transplantation has been well documented.^[10–12]^ Moreover, compared with the general population, CRC that are diagnosed in kidney transplant patients often display a more aggressive behavior, and the aggressive behavior is characterized by an earlier age of cancer diagnosis,^[13]^ a more advanced cancer stage (American joint committee on cancer stage > II),^[14]^ and a lower 5-year survival.^[13,15]^ Interestingly, a significantly reduced risk of rectal cancer was observed in the transplant recipients when separated from colon cancer,^[16,17]^ and it seems that the elevated risk of CRC was driven by excess of proximal colon cancer.^[17]^ One possible explanation to these results might be that transplant recipients were screened more frequently than the general population primarily through sigmoidoscopy, which did not reach the proximal colon, highlighting the importance of colonoscopy for CRC screening. There have been strong evidences that long-term immunosuppression increases the risk of CRC after kidney transplantation.^[11]^ The use of some specific immunosuppressive agents like azathioprine and calcineurin inhibitor (CNI) including tacrolimus and cyclosporine were also proposed to be associated with higher incidence of post-transplant malignancy.^[17,18]^ The patient in our report developed advanced rectal cancer (pT3N1M0) 4 years after kidney transplantation at an age of 50 years. Based on the general rule, tumors detected within the first 12 months after transplantation are correlated with pre-existed condition. Despite the fact that he had not received colonoscopic surveillance before or at the time of transplantation, we considered the diagnosed rectal cancer as the de novo malignancy. While, long-term exposure to CNI-based triple immunosuppressive agents together with an absence of colonscopic surveillance are speculated to be the 2 major hazard factors for occurrence of rectal cancer in our case.^[18]^

It is generally accepted that surgery plays a role in the treatment for CRC after transplantation. Several studies found surgeries exerted a positive effect on survival of transplant patients with CRC.^[14,19]^ Theoretically, these patients were supposed to be more susceptible to perioperative complications. However, Krysa^[20]^ assessed the outcome of 21 kidney transplant recipients undergoing elective colorectal surgery, and suggested the results were favorable, with no transplant rejection, low morbidity and mortality. Wisam^[21]^ also compared postoperative morbidity and oncologic outcome between patients with CRC in chronic immunosuppressive therapy and control groups. No significant difference was observed in wound infection, intra-abdominal abscess, anastomotic leak, urinary tract infection, or pneumonia, but lower in 3- and 5-year overall and disease-free survival. Consistently, several other reports demonstrated standard surgical treatment for CRC could be done safely in transplant recipients as long as the general condition and graft function were allowable.^[22,23]^

Regarding the relationship between option for timing of surgery and surgical outcomes, Lee^[24]^ found kidney transplant recipients undergoing colorectal resection <1 year of transplant had a higher perioperative mortality rate than those with grafts >1 year, likely due to more emergent surgeries in the early post-transplant period. Emergent colorectal surgery in kidney transplant patients was reported to have a significant risk of anastomotic leak; moreover, the overall major complication rate after emergent surgery was 81%, much higher than 19% of that after elective surgery.^[20]^ Therefore, emergent surgery for CRC in transplant recipients is not recommended considering its worse surgical outcomes.

Compared with conventional open surgery, laparoscopic surgery for rectal cancer possesses comparable oncologic outcomes, and remarkable short-term advantages, particularly, a lower intra-postoperative complication rate.^[2,3]^ Immunosuppression is known to delay wound healing, increase infection risk, and lead to hemorrhage, anemia, as well as renal failure,^[25]^ which may be a bigger problem to open surgery. Therefore, transplant recipients seem to benefit more from the minimal access approach. Alasari^[26]^ evaluated short- and long-term outcomes of minimally invasive (laparoscopic and robotic) colorectal resection in 10 kidney transplant recipients with CRC between May 2007 and August 2012. Having observed a favorable result in operative time (192.5 ± 15 min), blood loss (30 ± 50 mL), and postoperative complication (2/10 minor complications), they proposed minimally invasive colorectal procedures could be considered as safe and feasible alternatives to open colorectal resection in kidney transplant patients. In our report, the patient underwent laparoscopic assisted low anterior resection and prophylactic loop transverse colostomy. We evaluate the outcome of surgery from 3 respects as follows.

### 3.1 Short-term outcomes

Laparoscopic resection for CRC in kidney transplant patients is technically feasible. Duration of our surgery was <2 h, and intraoperative blood loss was little. Analgesia pump was not used postoperatively. Except for the anastomotic hemorrhage, no complications occurred including wound or urinary tract infections, pneumonia, anastomotic leakage, and prolonged ileus. Passing flatus began early represented a fast recovery of intestinal function. The favorable outcome in our case provided a powerful support for the advantages of laparoscopic surgery in the treatment of transplant patients with CRC. Most concerns about the use of laparoscopic surgery in CRC focused on technical complexity and longer operative time.^[2]^ However, it was shown that duration of laparoscopic surgery decreased significantly with the number of interventions performed, accompanied with a significant reduction in postoperative morbidity as the surgeon gained more experience.^[27]^ Having performed 148 laparoscopic surgery for CRC in the high-risk elderly patients including 3 kidney and 1 heart transplant recipients from 2010 to 2012 in our institution, we accumulated abundant experience that wound complication was 3.3%, and no case of anastomotic leakage was identified.^[28]^ To prevent anastomotic leakage, temporary diverting ostomy has been recommended for those patients at high risks such as transplant recipients on immunosuppressive therapy,^[29]^ which is our routine method to protect anastomosis. The most common stoma options are the loop transverse colostomy or loop ileostomy. For our patient, his graft was located in the right lower quadrant with severe adhesions from previous surgery around, which may easily get injured during the loop ileostomy. In addition, much more fluid loss after loop ileostomy than loop transverse colostomy leads to a higher incidence of renal insufficiency.^[30]^ For these reasons, loop transverse colostomy seemed to be a better option in this case. Anastomotic bleeding after laparoscopic rectal surgery is not rare. The use of a circular side stapling technique in laparoscopic low anterior resection for rectal cancer proved to be safe and did not increase the risk of anastomotic complications.^[31]^ Possible reason for bleeding in our case was likely to be attributed to the lower location of tumor.^[32]^

### 3.2 Long-term oncologic outcome

Laparoscopic resection for CRC in kidney transplant patients is oncologically safe. Oncologic outcome is usually measured by the extent of resection, disease-free survival and overall survival. Curative extent of resection represents radical tumor removal with negative margins, TME, and a sufficient number of lymph nodes (>12).^[33]^ During our surgery, resection achieved adequate range of intestinal segment and total mesorectum. In addition, 14 lymph nodes were harvested, which fulfilled the standard of radical operation. Similar outcomes could be achieved by Rivas^[8]^ during the laparoscopic resection for colon cancer in transplant patients, as long as the allograft was placed in the contralateral side of the colon resection. As for disease-free survival and overall survival, our patient was alive without recurrence and metastasis after 4 months of follow-up. Further follow-up is needed to evaluate his long-term survival.

### 3.3 Graft function and immunosuppression modification

In this case, 2 trocars were placed away from the incision scar of the transplant surgery and the position of graft. Slightly lower pneumoperitoneum pressure during the surgery was maintained to preserving an adequate allograft function. Immunosuppressive therapy was not stopped perioperatively to avoid danger of rejection, and postoperative serum creatinine levels stayed within the normal limits. In recent years, immunosuppressive medication modification including CNI-free regimens, substitution by mammalian target of rapamycin inhibitors or reduction in dosage of immunosuppression has been utilized as a treatment after cancer diagnosis in some transplant patients.^[34,35]^ It has been speculated that the use of rapamycin, instead of CNI might reduce the recurrence of cancer in transplant patients.^[36]^ In a case of adenocarcinoma in the stage of III B, as in our patient, switching immunosuppression regimen from cyclosporin to rapamycin might be helpful for his survival after surgery.

Based on the favorable oncologic outcome and low operative complications, laparoscopic assisted resection might be a preferred option for transplant patients with CRC. Indeed, decisions regarding surgical approach should also take into consideration of surgeon experience, tumor stage, potential contraindications, and patient expectations.

In summary, we have reported a case of a patient after kidney transplantation, in whom laparoscopic assisted low anterior resection for advanced rectal cancer had been performed successfully. The de novo rectal cancer was speculated to associate with long-term exposure to CNI-based immunosuppressive agents and an absence of colonscopic surveillance. We believe laparoscopic surgery for CRC in transplant recipients is technically feasible and oncologically safe, which could be a preferred option of surgical procedure in the near future.
